# Concentration Characteristics and Correlations with Other Pollutants of Atmospheric Particulate Matter as Affected by Relevant Policies

**DOI:** 10.3390/ijerph20021051

**Published:** 2023-01-06

**Authors:** Hong Song, Yuhang Dong, Jiayu Yang, Xin Zhang, Xingxin Nie, Yuesheng Fan

**Affiliations:** 1 School of Management, Xi’an University of Architecture and Technology, Xi’an 710055, China; 2 School of Resources Engineering, Xi’an University of Architecture and Technology, Xi’an 710055, China; 3 School of Environmental and Municipal Engineering, Xi’an University of Architecture and Technology, Xi’an 710055, China; 4 School of Building Services Science and Engineering, Xi’an University of Architecture and Technology, Xi’an 710055, China

**Keywords:** particulate matter, distribution characteristics, Xi’an, correlation, model

## Abstract

With the increase in global environmental pollution, it is important to understand the concentration characteristics and correlations with other pollutants of atmospheric particulate matter as affected by relevant policies. The data presented in this paper were obtained at monitoring stations in Xi’an, China, in the years from 2016 to 2020, and the spatial distribution characteristics of the mass and quantity concentrations of particulate matter in the atmosphere, as well as its correlation with other pollutants, were analyzed in depth. The results showed that the annual average concentrations of PM_10_ and PM_2.5_ decreased year by year from 2016 to 2020. The annual concentrations of PM_2.5_ decreased by 20.3 μg/m^3^, and the annual concentrations of PM_10_ decreased by 47.3 μg/m^3^. The days with concentrations of PM_10_ exceeding the standards decreased by 82 days, with a decrease of 66.7%. The days with concentrations of PM_2.5_ exceeding the standards decreased by 40 days, with a decrease of 35.4%. The concentration values of PM_10_ and PM_2.5_ were roughly consistent with the monthly and daily trends. The change in monthly concentrations was U-shaped, and the change in daily concentrations showed a double-peak behavior. The highest concentrations of particulate matter appeared at about 8:00~9:00 am and 11:00 pm, and they were greatly affected by human activity. The proportion of particles of 0~1.0 μm decreased by 1.94%, and the proportion of particles of 0~2.5 μm decreased by 2.00% from 2016 to 2020. A multivariate linear regression model to calculate the concentrations of the pollutants was established. This study provides a reference for the comprehensive analysis and control of air pollutants in Xi’an and even worldwide.

## 1. Introduction

With the continuous worsening of complex and intersecting air pollution problems in recent years, people have been paying more attention to a series of problems caused by air pollution [1]. The serious excess of particulate matter and other gaseous pollutants in the atmosphere not only leads to the decline in atmospheric visibility but also seriously affects routine travel and transportation [2] and even causes harm to the human body to various extents [3,4]. The relevant literature shows that particles of different sizes can cause respiratory diseases, infectious diseases, etc. [5]. The large-scale spread of COVID-19 (corona virus disease 2019) has brought unprecedented catastrophic effects on the life and health of people all over the world [6] and has caused numerous deaths. In addition, toxic and harmful gases, viruses, and bacteria attach to the surface of particulate matter [7], which allows them to enter the human body and causes a chain reaction whose effects can be life threatening. Therefore, how to create an appropriate and healthy living environment has unanimously become the priority of nations worldwide.

Therefore, many countries around the world have issued a series of relevant policies and standards to limit the concentration of the emissions of various pollutants in the atmosphere [8,9,10]. The determination of concentration distribution characteristics and the source analysis of pollutants in the atmosphere have gradually become the focus of scholars at home and abroad at the same time [11,12,13,14,15]. Research mainly focuses on the composition of particulate matter [11], human health effects [12], correlation studies on different pollutants [13,14], and the control of particulate matter pollution [15]. In addition, how pollutants are measured, which are the most polluted areas, which activities are the most polluting, and what is the impact of external factors on the accuracy of measurement techniques are also hot points [16,17,18], with an example being the performance of low-cost sensors for air quality monitoring [16]. However, there have been relatively few studies on the changes in the concentration distribution of particulate matter and its correlation with other major pollutants in recent years. Current research on air pollutants in China is also limited to certain areas, such as Nanjing [19], Yunnan [20], Beijing [21], and other places; meanwhile, there have been relatively few recent studies on northwest inland cities. In addition, there is a significant difference between the north and the south in China, and the particle concentration distributions in different regions show even greater differences [22]. Many different countries and regions around the world have introduced stricter control measures and control methods for monitoring pollutants in the atmosphere and have also strengthened energy adjustment measures and increased implementation efforts in recent years. All of these factors will likely lead to changes in the concentration distribution characteristics of pollutants in the atmosphere and the correlations among pollutants; there might even be unexpected results. However, there are still relatively few relevant studies, and the overall research effort is slightly insufficient. In particular, there is a serious lack of research on the concentration distribution of pollutants in typical northwest cities in China under the relevant control measures employed in recent years.

The spatio-temporal distribution of the quantity and mass concentrations of particulate matter in the atmosphere in Xi’an, as well as the correlations with other pollutants, were analyzed in this paper using monitoring data and recorded data from 2016 to 2020. This study could help stakeholders to clearly understand the change characteristics of atmospheric concentration distributions in the five years considered as affected by relevant policies; furthermore, it could also provide a reference for the comprehensive management of and improvement in the atmospheric environment across the country and the world.

## 2. Methods

Xi’an, with located longitude 107.40°~109.49° E and latitude 33.42°~34.45° N, was selected as the research area [23]. It is a long-established, old-civilization city with more than 5000 years of civilization history [23], and it has a large population. However, the atmospheric environmental conditions in Xi’an have become increasingly serious in recent years, and this has deeply affected people’s lives. To solve this problem effectively and rapidly, the government took a series of measures to protect the atmosphere in 2017, and these were rapidly implemented. Examples include urban development without coal, and coal-to-electricity and coal-to-gas transitions [24], and certain results were initially achieved. Therefore, we decided to select the atmospheric data of Xi’an of a recent five-year period (2016–2020) for in-depth research.

The data in this paper covered the period from 31 January 2016 to 31 December 2020 (provided by the tianqihoubao network (http://www.tianqihoubao.com/aqi/xian, accessed on 6 June 2021)) and included the daily average concentration values of SO_2_, NO_2_, PM_10_, PM_2.5_, O_3_, and CO. The hourly concentration values of six pollutants as recorded at monitoring stations in Xi’an were provided by Weather Network (http://www.tianqi.com/air/xian.html, accessed on 6 June 2021). GRIMM1.109 portable aerosol spectrometers (GRIMM Aerosol, Ainring, Germany) were used to measure the concentration of particles in the atmosphere. The particles ranging from 0.25 to 32 μm in diameter could be separated into 31 channels. Repeatability was 5%. The average concentration over 20 min was recorded, and the data were analyzed using mean values to reduce the experimental error. To ensure the validity of data statistics, we report the Chinese standards for reference [25,26,27], as this allows one to analyze data more efficiently. The Chinese standards establish daily average concentrations of 35~75 μg/m^3^ PM_2.5_, 50~150 μg/m^3^ PM_10_, 50~150 μg/m^3^ SO_2_, 80 μg/m^3^ NO_2_, 4 mg/m^3^ CO, and 160~200 μg/m^3^ O_3_ (1-h values in the case of O_3_) [25].

## 3. Results and Discussion

### 3.1. Annual Concentrations of Particulate Matter

**Figure 1:**
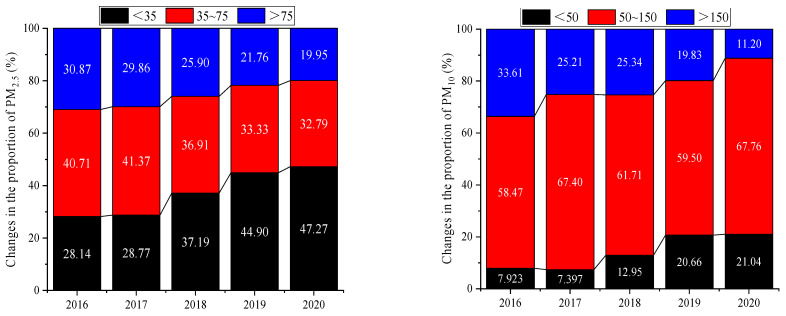
Changes in PM_2.5_ and PM_10_ concentrations from 2016 to 2020.

Figure 1 shows that the changes in the proportions of PM_2.5_ and PM_10_ concentrations per year followed the same trend. The proportions of PM_2.5_ concentrations lower than 35 μg/m^3^ and of PM_10_ concentrations lower than 50 μg/m^3^ were relatively low, and the proportions increased gradually over time. The proportions of PM_2.5_ concentrations between 35 and 75 μg/m^3^ and of PM_2.5_ concentrations higher than 75 μg/m^3^ gradually decreased. On the other hand, the proportions of PM_10_ concentrations between 50 and 150 μg/m^3^ showed a gradually increasing trend, with a fluctuating and dominant position. The proportions of PM_10_ concentrations higher than 150 μg/m^3^ gradually decreased, and the trend was the same as that of PM_2.5_ concentrations. It can be clearly seen from the graphs that the proportion of days with low concentrations increased and that the proportion of days with concentrations exceeding the standards decreased, which indicates that the air quality in Xi’an gradually improved and the concentrations of particulate matter in the atmosphere gradually decreased. More specifically, the concentrations of particulate matter changed significantly from 2017 to 2018. The proportions of PM_2.5_ concentrations lower than 35 μg/m^3^ increased by 8.42%. The main reasons were that a series of measures were taken for controlling air pollution and that relevant energy production methods were adjusted, which had an obvious effect on the concentration of particulate matter emissions. The specific parameters are shown in Table 1.

It can be seen from the table that the annual average concentrations of PM_2.5_ and PM_10_ gradually decreased from 2016 to 2020. The annual concentrations of PM_2.5_ decreased by 20.3 μg/m^3^, and the annual concentrations of PM_10_ decreased by 47.3 μg/m^3^. The annual average concentrations of PM_10_ were 1.96 times, 1.86 times, 1.70 times, 1.46 times, and 1.29 times that of the secondary standard from 2016 to 2020, and 3.43 times, 3.26 times, 2.97 times, 2.56 times, and 2.25 times that of the primary standard [25]. The annual average concentrations of PM_2.5_ were 2.02 times, 2.06 times, 1.73 times, 1.65 times, and 1.44 times that of the secondary standard and 4.71 times, 4.81 times, 4.04 times, 3.85 times, and 3.36 times that of the primary standard [25]. The number of days with concentrations exceeding the standards showed a downward trend. The average number of days with concentrations of PM_10_ exceeding the standards was 84 days from 2016 to 2020, and the days with concentrations of PM_10_ exceeding the standards decreased by 82 days, with a decrease of 66.7%. The average number of days with concentrations of PM_2.5_ exceeding the standards was 94 days, and the days with concentrations of PM_2.5_ exceeding the standards decreased by 40 days, with a decrease of 35.4%. Therefore, the concentrations of particulate matter in the atmosphere in Xi’an in the five years considered were effectively controlled, and certain results also were achieved, but further in-depth management is still required.

### 3.2. Seasonal Concentrations of Particulate Matter 

**Figure 2:**
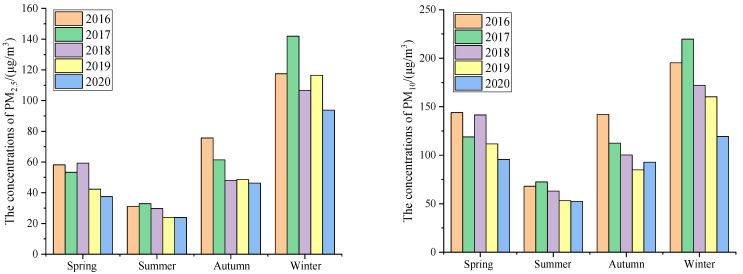
Seasonal changes in PM_2.5_ and PM_10_ concentrations from 2016 to 2020.

The seasonal average statistics according to the climatic conditions of the Chinese region were defined for spring (March to May), summer (June to August), autumn (September to November), and winter (December to February) [13,28,29]. The average concentrations of particulate matter in different seasons are shown in Figure 2.

Figure 2 shows that the seasonal average concentrations of different particles in the five years considered presented good similarity, but there were still certain differences in different seasons. The concentrations of PM_2.5_ followed the order of winter > autumn > spring > summer, with the five-year average concentrations being 115.2 μg/m^3^, 56.0 μg/m^3^, 50.1 μg/m^3^, and 28.3 μg/m^3^, respectively. The concentrations in winter were 2.06 times higher than those in autumn, 2.30 times higher than those in spring, and 4.07 times higher than those in summer. The concentrations of PM_10_ followed the order of winter > spring > autumn > summer, with the five-year average concentrations being 173.3 μg/m^3^, 122.4 μg/m^3^, 106.5 μg/m^3^, and 61.8 μg/m^3^, respectively. The concentrations in winter were 1.42 times higher than those in spring, 1.63 times higher than those in autumn, and 2.81 times higher than those in summer. Overall, it was observed that the concentrations of particulate matter were the highest in winter and the lowest in summer, which is consistent with the literature [22].

### 3.3. Monthly Concentrations of Particulate Matter

**Figure 3:**
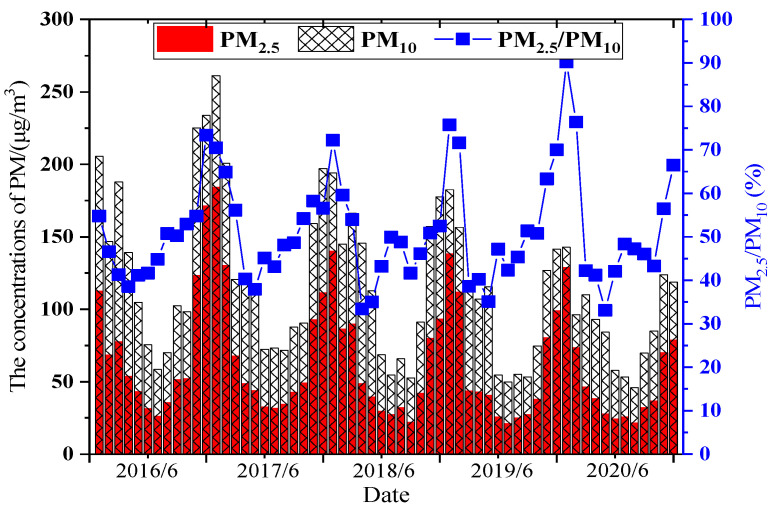
Monthly changes in PM_2.5_ and PM_10_ concentrations from 2016 to 2020.

The average concentrations of particulate matter in different months are shown in Figure 3.

Figure 3 shows that the monthly average concentrations of PM_2.5_ and PM_10_ all showed a U-shaped trend. The average monthly concentrations of PM_2.5_ and PM_10_ were the lowest in July and October, while the average monthly concentration of PM_2.5_ and PM_10_ were the highest in December and January. It could also be seen that the concentrations of particulate matter in the same months from 2016 to 2020 showed a gradually decreasing trend. The concentrations of PM_2.5_ rose rapidly from the lowest value in July to December and then began to decrease in spring. The concentrations of PM_10_ still had a high value in spring, which might have been related to the frequent occurrence of dusty weather in spring [23]. The average annual proportions of PM_2.5_/PM_10_ from 2016 to 2020 were 49.2%, 52.0%, 48.9%, 52.6%, and 52.8%, respectively. The atmospheric particles in Xi’an are mainly fine particles, and it is still necessary to further study the source of fine particulate matter.

### 3.4. Daily Concentrations of Particulate Matter

**Figure 4:**
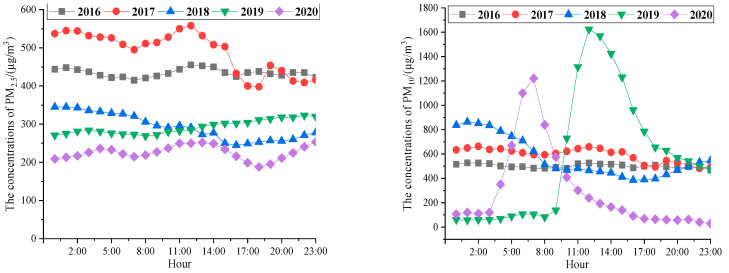
Daily changes in PM_2.5_ and PM_10_ concentrations from 2016 to 2020.

The five days with the largest concentrations of PM_2.5_ were adopted in this paper, namely, 20 December 2016; 5 January 2017; 15 January 2018; 6 January 2019; and 25 January 2020. For PM_10_, these were 20 December 2016; 5 January 2017; 3 December 2018; 12 May 2019; and 26 March 2020. The time period was from 0:00 to 24:00. The average distributions of different daily concentrations of particulate matter are shown in Figure 4.

Figure 4 shows that the hourly average distribution trends of PM_2.5_ and PM_10_ were consistent with a bimodal distribution. In addition, it can be seen that the maximum concentrations in 2018, 2019, and 2020, after the adoption of relevant measures, were much lower than the maximum concentrations in 2016 and 2017, before the adoption of measures. The concentrations of PM_2.5_ and PM_10_ in 2020 were relatively low, which indirectly showed that the phenomenon of high concentrations of particulate matter was controlled after the adoption of relevant measures. The concentrations at night were higher than those in the daytime. The main reason was the demand for heating in winter, which leads to an increase in the concentrations of particulate matter. Another reason was the influence of vertical movement in the atmosphere, which is not conducive to the spread of particles [22,23]. As a result, the concentrations of particulate matter reached the maximum at around 11 o’clock at night. The concentrations of particulate matter gradually increased from about 8:00 in the morning, which corresponds to the commuting peak, when pollutant emissions also reach the peak of the day. The concentrations of particulate matter gradually decreased in the afternoon. The main reasons were that solar radiation is stronger and the temperature of the environment is higher, which make turbulent exchange and diffusion of gases stronger. The atmosphere near the ground in the evening is more unstable, and the lowest concentrations of particulate matter appeared around 17:00. Human activity had a significant impact on the concentrations of particulate matter, and this conclusion is consistent with the conclusion given by Zhu Changlin [28], which verifies the validity of the results of this paper. However, the concentrations of PM_10_ on 12 May 2019 and 26 March 2020 changed significantly, which might have been related to local meteorological parameters or the environment on those days [30]. The specific parameters of typical days in Xi’an are shown in Table 2.

### 3.5. Quantity Concentrations of Atmospheric Particles

**Figure 5:**
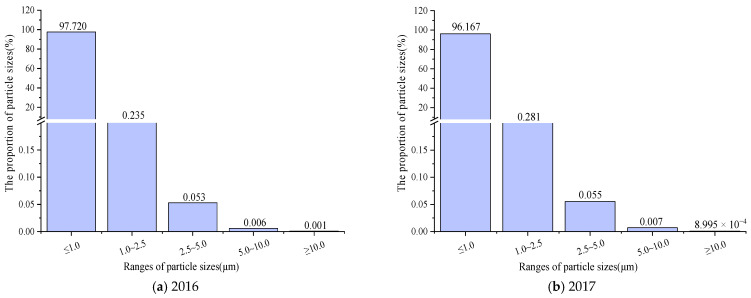

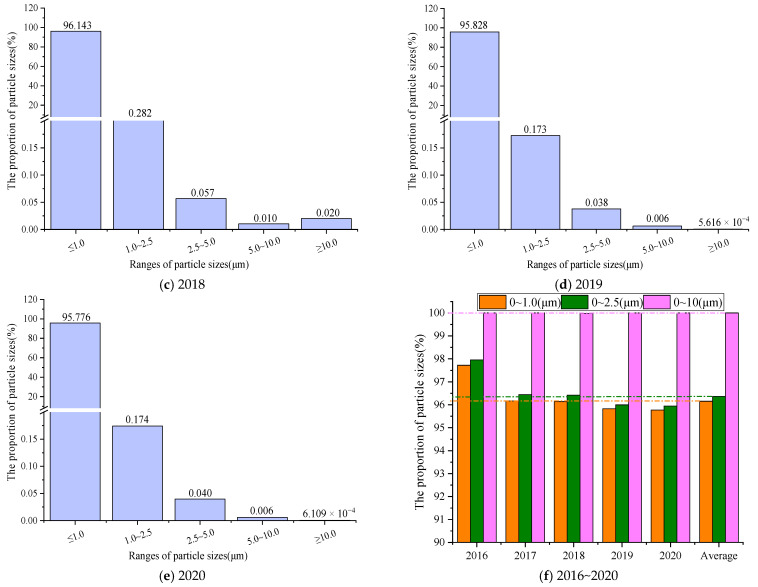
Distributions of outdoor atmospheric particle sizes (average temperature of 19.9 °C~25.5 °C; average humidity of 38.9~50.9%).

Outdoor atmospheric dust was used as the test dust source [31]. The atmospheric particle size distributions during the test period are shown in Figure 5.

Figure 5a–e shows the quantity distributions of particle sizes in the outdoor atmosphere during the test period. It can be seen that particles of sizes of 0~1.0 μm accounted for the vast majority from 2016 to 2020, i.e., about 97.72%, 96.17%, 96.14%, 95.83%, and 95.78%, respectively, with the proportion decreasing by 1.94%. Particles of sizes of 1.0~2.5 μm accounted for about 0.235%, 0.281%, 0.282%, 0.173%, and 0.174%, respectively, with the proportion decreasing by 0.061%. However, the proportion of particles of sizes above 2.5 μm was relatively small; this is consistent with the conclusions given in the literature, which verifies the validity of the results of this paper [22,23]. The changes in the 5 years considered were relatively insignificant for particles of sizes larger than 2.5 μm, but there was still a trend of decreasing concentrations.

Figure 5f shows that particles of an average particle size between 0 and 1.0 μm accounted for 96.16% of the total in the five years considered, while particles of sizes between 0 and 2.5 μm accounted for 96.37%, and particles of sizes between 0 and 10 μm accounted for about 100%. It is clear that in Xi’an, particles are mainly composed of fine particles; such particles can easily enter the respiratory tract and lungs of the human body, leading to diseases and death [32]. With the rapid implementation of measures for the control of atmospheric particulate matter concentrations, the proportion of particles of 0~1.0 μm decreased by 1.94%, and the proportion of particles of 0~2.5 μm decreased by 2.00%. The implementation of relevant measures and governance policies has achieved certain results, but it is still necessary to increase the purification effect on fine particles in the future to achieve the creation of a healthy environment.

### 3.6. Correlations between Particulate Matter and Other Major Pollutants

**Figure 6:**
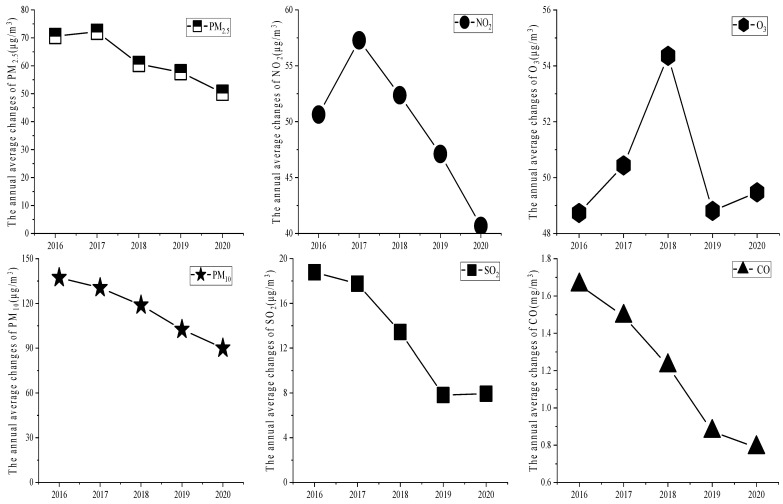
Trends of annual average changes in quantities of various pollutants.

Various gaseous pollutants in the atmosphere are important sources of secondary pollution of particulate matter [33]. Therefore, it is of great significance to study the correlations between the concentrations of particulate matter and other major gas pollutants to effectively control the concentrations of each pollutant. The annual average changes in PM_2.5_, PM_10_, CO, SO_2_, NO_2_, and O_3_ in Xi’an from 2016 to 2020 are shown in Figure 6.

Figure 6 shows that all the considered pollutants showed a downward trend in the five years under analysis. The annual average concentrations of NO_2_ were similar to those of PM_2.5_, which first increased and then decreased. The annual average concentrations of SO_2_ and CO showed a trend similar to that of PM_10_, and both showed a decreasing trend. In contrast, the annual average concentrations of O_3_ showed a different trend: they first increased, then decreased, and then increased again over time. The annual average concentrations of NO_2_ decreased by 16.6 μg/m^3^ from 2016 to 2020; those of SO_2_ decreased by 11.0 μg/m^3^; and those of CO decreased by 0.873 mg/m^3^. Furthermore, the overall annual concentrations of O_3_ decreased by 5.6 μg/m^3^. The correlations among pollutants are shown in Table 3.

It can be seen from the table that the average correlation between PM_10_ and PM_2.5_ in the five years considered was 0.740, which means that they were highly correlated [22,34]. The average correlation between SO_2_ and PM_2.5_ was 0.545, and the average correlation between SO_2_ and PM_10_ was 0.464, with both being moderate correlations. The average correlation values between NO_2_, and PM_2.5_, PM_10_, and SO_2_ were 0.481, 0.448, and 0.512, respectively, which were also moderate correlations. The average correlation values between CO, and PM_2.5_, PM_10_, SO_2_, and NO_2_ were 0.831, 0.526, 0.600, and 0.456, respectively, indicating that CO was highly correlated with PM_2.5_ and moderately correlated with the other pollutants. The average correlation values between O_3_, and PM_2.5_, PM_10_, SO_2_, NO_2_, and CO were 0.288, 0.204, 0.305, 0.317, and 0.351, respectively, all of which were weak correlations. It can be seen that the concentrations of all pollutants decreased from 2016 to 2020 after the implementation of relevant measures and that the correlations among pollutants also decreased. The results show that the atmospheric environment in Xi’an demonstrated great improvements after the implementation of relevant measures; however, long-term governance management is still needed.

It is practical to establish a multiple linear regression model for estimation. Here, it was calculated according to Formula (1).

(1) $$Y_{i}=\beta_{0}+\beta_{1}X_{1}+\beta_{2}X_{2}+\cdot \cdot \cdot \cdot \cdot +\beta_{k}X_{k}$$

where $X_{1},X_{2},\cdot \cdot \cdot X_{k}$ are the average daily concentration values of pollutants, and $\beta_{0},\beta_{1},\cdot \cdot \cdot \beta_{k}$ are the coefficients.

The average data of the daily average concentrations of various pollutants from 2016 to 2020 were used for the analyses, and the collated concentrations of pollutants such as CO, SO_2_, NO_2_, and PM_10_ were substituted into Formula (1) for the calculation. The multiple regression formula of PM_2.5_ is presented in Formula (2).

(2) $$Y=0.265X_{1}+0.078X_{2}-0.078X_{3}+63.9X_{4}-42.7$$

**Figure 7:**
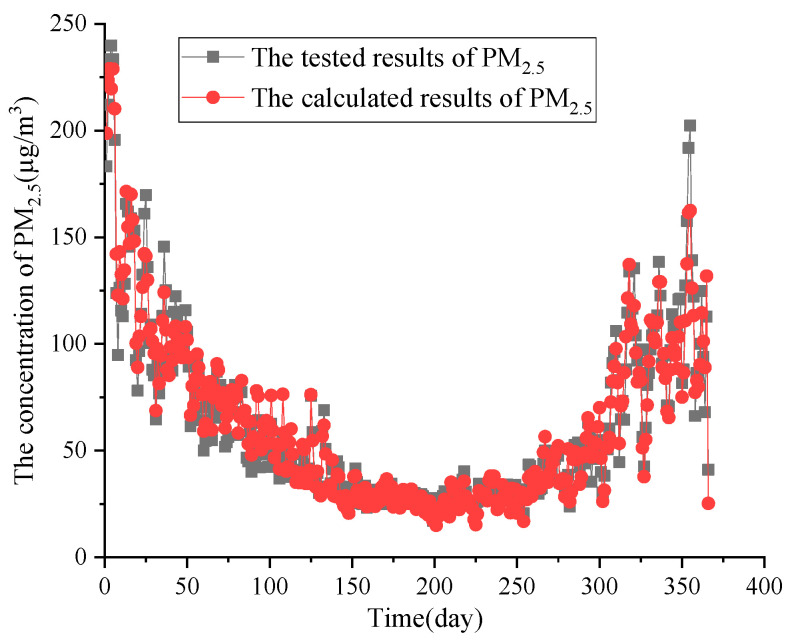
Comparison of calculated results and actual data of PM_2.5_.

The correlation coefficient (R) was 0.961, and the decision coefficient (R^2^) was 0.961; the regression equation had an obvious effect. The daily average concentrations of CO, SO_2_, NO_2_, and PM_10_ were substituted into Formula (2). The comparison of the actual PM_2.5_ data and the calculated results are shown in Figure 7.

The results show that the calculated results and actual data of the daily average concentrations of various pollutants from 2016 to 2020 were in good agreement, with the model showing a good prediction effect. However, there was a large fluctuation between PM_2.5_ results and data in spring and winter, while the relative consistency between them in summer and autumn was higher. There was a certain deviation in the prediction effect, but overall, it still showed good agreement [23]. Therefore, it was concluded that the daily average concentrations of pollutants in the atmosphere could be effectively predicted and monitored using the multiple linear regression model (Equation (2)). This model is of great significance to understand and analyze the spatio-temporal concentration distribution characteristics and correlations with other pollutants of atmospheric particulate matter as affected by relevant policies employed recently [35]. 

## 4. Conclusions

In this paper, the spatial distribution characteristics of the mass and quantity concentrations of particulate matter in the atmosphere, as well as its correlations with other pollutants as affected by relevant policies, were analyzed in depth. As a typical northwest city, Xi’an, China, was selected as the research area; the period considered in this study comprised the years from 2016 to 2020. We drew the following conclusions:

1. The annual average concentrations of PM_10_ and PM_2.5_ decreased year by year from 2016 to 2020. The annual concentrations of PM_2.5_ decreased by 20.3 μg/m^3^, and the annual concentrations of PM_10_ decreased by 47.3 μg/m^3^. The decrease in PM_10_ concentrations was greater than that in PM_2.5_. The days with concentrations of PM_10_ exceeding the standards decreased by 82 days, with a decrease of 66.7%. The days with concentrations of PM_2.5_ exceeding the standards decreased by 40 days, with a decrease of 35.4%.
2. The concentration values of PM_10_ and PM_2.5_ were roughly consistent with the monthly and daily trends. The distribution of PM_10_ was higher in winter and lower in summer. The distribution of PM_2.5_ was higher in winter and lower in autumn. The change in monthly concentration was U-shaped, with the lowest being in July and the highest being in December. The changes in daily concentrations showed a double-peak behavior. The highest concentrations of particulate matter appeared at about 8:00~9:00 am and 11:00 pm, and they were greatly affected by human activity.
3. The atmospheric particles in Xi’an were mainly fine particles. The proportion of particles of 0~1.0 μm decreased by 1.94%, and the proportion of particles of 0~2.5 μm decreased by 2.00% from 2016 to 2020. This study shows that the atmospheric environment in Xi’an demonstrated great improvements after relevant measures were implemented. A multivariate linear regression model to calculate the concentrations of pollutants was also established in this paper. This study provides a reference for the comprehensive analysis and control of air pollutants in Xi’an and even worldwide.

## Figures and Tables

**Table 1:** Parameter statistics of PM_10_ and PM_2.5_ from 2016 to 2020.

Content	Year	Average (μg/m^3^)	Max (μg/m^3^)	Min (μg/m^3^)	Number of Samples (Days)	Days with Concentrations Exceeding the Standards (Days)	Average Exceeding Concentration (μg/m^3^)	Percentage of Days with Concentrations Exceeding the Standards (%)
PM_10_ (μg/m^3^)	2016	137.3	501	24	366	123	238.8	33.6
	2017	130.5	591	17	365	92	255.4	25.2
	2018	118.9	568	20	363	92	229.5	25.3
	2019	102.5	576	10	363	72	212.5	19.8
	2020	90.0	297	16	366	41	190.0	11.2
PM_2.5_ (μg/m^3^)	2016	70.6	434	11	366	113	139.3	30.9
	2017	72.1	490	8	365	109	147.4	29.9
	2018	60.7	292	9	363	94	129.1	25.9
	2019	57.7	292	5	363	79	141.2	21.8
	2020	50.3	225	6	366	73	118.1	19.9

**Table 2:** Statistics of PM_10_ and PM_2.5_ on typical days from 2016 to 2020.

Content	Date	Quality Level	Daily Concentration (μg/m^3^)	Concentration Range (μg/m^3^)	Weather	Wind Direction	Wind Level
PM_2.5_ (μg/m^3^)	20 December 2016	Heavily polluted	434	415–455	Hazy	Northeasterly	1–2
	5 January 2017	Heavily polluted	490	398–558	Drizzly	East wind	1–2
	15 January 2018	Heavily polluted	292	245–345	Foggy	Southwesterly	2
	6 January 2019	Heavily polluted	292	271–320	Hazy	North wind	1–2
	25 January 2020	Heavily polluted	225	188–253	Drizzly	East wind	3–4
PM_10_ (μg/m^3^)	20 December 2016	Heavily polluted	501	470–528	Hazy	Northeasterly	1–2
	5 January 2017	Heavily polluted	591	483–663	Drizzly	East wind	1–2
	3 December 2018	Heavily polluted	568	386–864	Hazy	Southwesterly	1–2
	12 May 2019	Heavily polluted	576	57–1624	Gloomy	Northwesterly	3–4
	26 March 2020	Moderately polluted	297	29–1221	Cloudy	Northwesterly	3–4

**Table 3:** Correlations among air pollutants in the atmosphere from 2016 to 2020.

Year	Pollutant	Pollutant					
		PM_2.5_	PM_10_	SO_2_	NO_2_	CO	O_3_
2016	PM_2.5_	1					
	PM_10_	0.799	1				
	SO_2_	0.509	0.547	1			
	NO_2_	0.562	0.552	0.461	1		
	CO	0.709	0.677	0.746	0.54	1	
	O_3_	0.39	0.327	0.414	0.308	0.504	1
2017	PM_2.5_	1					
	PM_10_	0.811	1				
	SO_2_	0.622	0.51	1			
	NO_2_	0.578	0.437	0.615	1		
	CO	0.903	0.646	0.692	0.556	1	
	O_3_	0.261	0.2	0.32	0.326	0.37	1
2018	PM_2.5_	1					
	PM_10_	0.694	1				
	SO_2_	0.664	0.433	1			
	NO_2_	0.574	0.394	0.57	1		
	CO	0.854	0.432	0.702	0.57	1	
	O_3_	0.268	0.167	0.221	0.342	0.271	1
2019	PM_2.5_	1					
	PM_10_	0.774	1				
	SO_2_	0.42	0.346	1			
	NO_2_	0.425	0.386	0.523	1		
	CO	0.842	0.481	0.459	0.399	1	
	O_3_	0.311	0.196	0.404	0.373	0.337	1
2020	PM_2.5_	1					
	PM_10_	0.622	1				
	SO_2_	0.509	0.486	1			
	NO_2_	0.265	0.47	0.395	1		
	CO	0.847	0.396	0.401	0.215	1	
	O_3_	0.210	0.129	0.168	0.235	0.274	1

## Data Availability

The study did not report any data.
